# A Mobile Health Wallet for Pregnancy-Related Health Care in Madagascar: Mixed-Methods Study on Opportunities and Challenges

**DOI:** 10.2196/11420

**Published:** 2019-03-05

**Authors:** Nadine Muller, Peter Martin Ferdinand Emmrich, Elsa Niritiana Rajemison, Jan-Walter De Neve, Till Bärnighausen, Samuel Knauss, Julius Valentin Emmrich

**Affiliations:** 1 Heidelberg Institute of Global Health Medical Faculty and University Hospital University of Heidelberg Heidelberg Germany; 2 Department of Infectious Diseases and Pulmonary Medicine Charité-Universitätsmedizin Berlin, corporate member of Freie Universität Berlin, Humboldt-Universität zu Berlin, and Berlin Institute of Health Berlin Germany; 3 Biosciences Eastern and Central Africa Hub International Livestock Research Institute Nairobi Kenya; 4 Department of Global Health and Population Harvard T.H. Chan School of Public Health Boston, MA United States; 5 Africa Health Research Institute Mtubatuba, KwaZulu-Natal South Africa; 6 Department of Experimental Neurology and Center for Stroke Research Charité-Universitätsmedizin Berlin, corporate member of Freie Universität Berlin, Humboldt-Universität zu Berlin, and Berlin Institute of Health Berlin Germany

**Keywords:** pregnancy, maternal health services, healthcare financing, cell phone, mobile applications, telemedicine, maternal mortality, health expenditures, marketing of health services, developing countries, Madagascar

## Abstract

**Background:**

Mobile savings and payment systems have been widely adopted to store money and pay for a variety of services, including health care. However, the possible implications of these technologies on financing and payment for maternal health care services—which commonly require large 1-time out-of-pocket payments—have not yet been systematically assessed in low-resource settings.

**Objective:**

The aim of this study was to determine the structural, contextual, and experiential characteristics of a mobile phone–based savings and payment platform, the Mobile Health Wallet (MHW), for skilled health care during pregnancy among women in Madagascar.

**Methods:**

We used a 2-stage cluster random sampling scheme to select a representative sample of women utilizing either routine antenatal (ANC) or routine postnatal care (PNC) in public sector health facilities in 2 of 8 urban and peri-urban districts of Antananarivo, Madagascar (Atsimondrano and Renivohitra districts). In a quantitative structured survey among 412 randomly selected women attending ANC or PNC, we identified saving habits, mobile phone use, media consumptions, and perception of an MHW with both savings and payment functions. To confirm and explain the quantitative results, we used qualitative data from 6 semistructured focus group discussions (24 participants in total) in the same population.

**Results:**

59.3% (243/410, 95% CI 54.5-64.1) saved toward the expected costs of delivery and, out of those, 64.4% (159/247, 95% CI 58.6-70.2) used household cash savings for this purpose. A total of 80.3% (331/412, 95% CI 76.5-84.1) had access to a personal or family phone and 35.7% (147/412, 95% CI 31.1-40.3) previously used Mobile Money services. Access to skilled health care during pregnancy was primarily limited because of financial obstacles such as saving difficulties or unpredictability of costs. Another key barrier was the lack of information about health benefits or availability of services. The general concept of an MHW for saving toward and payment of pregnancy-related care, including the restriction of payments, was perceived as beneficial and practicable by the majority of participants. In the discussions, several themes pointed to opportunities for ensuring the success of an MHW through design features: (1) intuitive technical ease of use, (2) clear communication and information about benefits and restrictions, and (3) availability of personal customer support.

**Conclusions:**

Financial obstacles are a major cause of limited access to skilled maternal health care in Madagascar. An MHW for skilled health care during pregnancy was perceived as a useful and desirable tool to reduce financial barriers among women in urban Madagascar. The design of this tool and the communication strategy will likely be the key to success. Particularly important dimensions of design include technical user friendliness and accessible and personal customer service.

## Introduction

### Background

Despite widespread political commitment to improve accessibility and utilization of skilled care during pregnancy by user fee exemption policies, out-of-pocket payments (OPP) remain the predominant mode of health care financing in several sub-Saharan countries [1]. However, the costs for skilled care frequently exceed the savings or assets that can be accessed at one time by a low-income household, potentially leading to medical impoverishment [2-4]. Besides selling assets or receiving emergency funds or loans by family or friends, personal or household savings can help protect from the financial burden of health shocks [1,5]. However, setting money aside on a regular basis to save for health care is difficult for the poor, who often face the need for imminent expenses, which undermines long-term saving goals [6]. Therefore, expectant mothers from low-income households in sub-Saharan Africa (SSA) often do not seek skilled birth attendance or emergency obstetric care to avoid the large costs associated with delivery [7].

Within the last decade, mobile phone ownership has grown exponentially, and mobile communication has become pervasive in SSA. Today, more than 70% of worldwide mobile phone subscriptions come from low- and middle-income countries (LMICs) with more than 74 subscriptions per 100 people in SSA in 2016 [8,9]. In the footsteps of this mobile phone revolution have followed mobile payment systems, colloquially known as Mobile Money (MM), which commonly utilize low-tech systems such as unstructured supplementary service data to enable financial transactions without the need for a bank account. MM is suitable for virtually all widely used handsets independent of smartphone capability or internet access, and it allows subscribers to send, save, and receive funds on a digital platform held by a mobile operator. Funds and transactions are typically denoted in the national currency, allowing saving with little purchasing power volatility and transactions without conversion fees. Cash can be converted into electronic value (and vice versa) at retail stores or agents [10]. With almost 80% of adults in SSA not having access to formal banking services, economies are increasingly relying on mobile payment systems [11]. In turn, this allows financial inclusion of households with low transaction volumes or limited access to the formal banking system [12].

Leveraging this technological development, mobile payment–based hospital insurance or a savings mechanism enables low-income households to set aside funds exclusively for health care. In Kenya, an electronic health savings account including e-vouchers and microinsurance schemes to improve health care access was launched in 2008 [13,14]. In addition, a mobile phone–based payment and savings platform allowing clients to send, save, and spend funds specifically for medical treatment in contracted health facilities has been commercially available in Kenya since 2016 [15]. To date, this system has reached 1.4 million registered users, and it has facilitated the payout of US $4.8 million [16]. Among the variety of medical conditions, maternal health care seems particularly well suited for a medical savings scheme as expenses are mostly predictable both in their timing and amount. However, these mechanisms have yet to be systematically applied to maternal health care savings.

In Madagascar, a low-income country with a maternal mortality rate of 353 per 100,000 [17,18], financial obstacles are a major cause for a lack of access to basic health care during pregnancy, with 44% of pregnant women not seeking skilled birth attendance [19,20]. Although Madagascar implemented a user fee exemption policy for maternal health care services in 2008, out-of-pocket costs for routine drugs and laboratory tests or hospitalizations remain a major obstacle for seeking skilled care [21]. Experimental introduction of free-at-point-of-care services in rural southeastern Madagascar in 2014 increased the use of formal health care by 65%, with antenatal visits increased by 25% [22]. The following year, a government policy aiming to establish universal health insurance coverage created a *basket fund* pooling public, donor, and private contributions to remove user fees [23]. This has helped to reduce out-of-pocket spending on health care from 31% in 2013 to 22% in 2015 [24].

Nevertheless, many health care providers remain unable to meet their costs without charging user fees. Less than 6% of Madagascans have a formal bank account, and the risk of impoverishing health expenditures as a result of pregnancy is high [18,25]. However, in parallel, many countries in SSA have undergone rapid digitization. In Madagascar, the mobile phone subscription rate increased 14-fold over the past decade, from less than 3 subscriptions per 100 people in 2005 to 42 subscriptions per 100 people in 2016 [8]. These figures suggest that mobile phones may be a promising tool to improve financial access to skilled health care delivery. However, little is known about current saving habits, use of mobile phones, and mobile payment systems among pregnant women in Antananarivo, particularly those from low-income households.

### Objectives

This mixed-methods study is among the first to identify the major opportunities and challenges related to the implementation of a Malagasy Ariary denoted mobile phone–based payment and savings platform, the so-called Mobile Health Wallet (MHW), for maternal health care in low-resource settings. A 2-stage cluster sampling design was used to randomly select 412 pregnant women and new mothers among basic health center visitors in urban areas of Antananarivo, Madagascar. To confirm, explain, and complete the quantitative survey results, we used qualitative data from 6 semistructured focus group discussions (FGDs) in the same population (24 participants in total). We assessed the following characteristics: (1) perceived obstacles to skilled birth attendance, (2) saving methods and habits, (3) usage of mobile phones and mobile payment systems, (4) perception of the usefulness and acceptability of MHW for maternal health care, and finally (5) media access and consumption for program information purposes.

## Methods

### Study Design

We used quantitative population-representative survey data and qualitative FGDs in a sequential explanatory mixed-methods design [26,27]. The quantitative part was used to measure the prevalence of multiple characteristics relevant among pregnant women in Madagascar. The qualitative data contributed to a deeper understanding of the respective findings. Integration of both methods occurred during the sampling and analysis stage. Participants were sampled from the same study populations for both the survey and FGDs. The analysis was integrated by using similar thematic coding for major barriers and facilitators of the MHW. Results were then triangulated for each of the themes across data sources.

### Study Setting

The study was conducted in Atsimondrano and Renivohitra, 2 out of 8 districts within the region of Analamanga, Madagascar. Both districts are mostly urban and include Antananarivo, the capital of Madagascar. These districts were chosen because of their high levels of mobile phone usage and good stability and coverage of mobile networks, allowing the implementation of an MM-based savings system.

### Quantitative Methodology

A quantitative survey was developed for the Madagascan context to collect general information on structural and contextual elements of MHW for skilled health care during pregnancy. To quantify the need for MHW, we elicited information about household income, use of mobile phones and mobile payment services, and savings methods and mechanisms during pregnancy.

#### Sampling

We conducted a 2-stage cluster-random sampling of women utilizing routine antenatal care (ANC) or routine postnatal care (PNC) in the public-sector health facilities in the Malagasy districts of Atsimondrano and Renivohitra. In the first stage, we randomly sampled public-sector health facilities in these 2 districts. To be eligible to be included in our sampling frame, the facilities needed to be approved by the Ministry of Health. Out of 38 public-sector health facilities in the 2 districts, we randomly selected 14 with equal probability. We excluded 4 as they were characterized by at least one of the following 3 exclusion criteria: (1) they refused to participate in this study, (2) they did not perform ANC at a minimum quantity (defined as less than 10 ANC visits per week in 2016), or (3) they were not reachable within 2 hours from the city center of Antananarivo by public transportation. The last exclusion criterion was applied to allow easy access by data collectors for this and follow-up studies. From the remaining 10 public-sector health facilities, 8 were so-called CSBs (*French: Centres de Santé de Base* or basic government-run health care centers) and 2 were referral hospitals. Most of the randomly selected facilities provided elective maternal health care only on specific days of the week. In the second stage, we thus randomly sampled 2 facilities for each calendar day during the period from November 29, 2017 to January 22, 2018 out of the sampling frame of those randomly selected clinics that were open on that day. All women utilizing ANC or PNC on the selected clinic days were eligible to participate in the survey interview and were approached for consent to participate. Sampling occurred on 23 working days in total. For sample size determination, a number of 83,297 pregnant women at a given moment in the study area was calculated on the basis of 2017’s population size of 1,851,024 for the districts Atsimondrano and Renivohitra and 4.5% of the women in the population were considered pregnant at a given moment as estimated by the Malagasy Ministry of Health (nonpublished data). Using the open source Web-based calculator OpenEpi Version 3 (Rollins School of Public Health, Emory University, USA), with a 95% CI and a 0.05 significance level, a sample size of 383 was deemed sufficient [28]. Out of a total of 416 eligible women, 412 gave consent and were included in the study. Participant recruitment was done by the data collectors while at a health facility.

#### Data Collection

A total of 3 experienced interviewers (2 women and 1 man), who were native speakers of Malagasy, were recruited from the Analamanga region as data collectors. Training included a 1-day standardized curriculum. Data collectors identified themselves to participants as independent researchers, who were not associated with the health care facility. Supervisors were present throughout the study period to ensure data quality and accuracy. Data collectors administered a paper-based questionnaire, which covered sociodemographic and economic characteristics, saving habits, mobile phone access, previous experience with mobile payment systems, and intended place to give birth, as well as multimedia consumption.

#### Data Analysis

We calculated descriptive statistics for the study sample including percentages, means, and 95% CI. The poverty status was estimated on the basis of the poverty line of US $1.90, referring to 2011 dollars, and taking into account US inflation (corresponding to US $2.03 in 2016). With a Purchasing Power Parity factor for Madagascar of 847.2 in 2016, the poverty line was calculated to be at 1719.8 Ariary (Ar) a day or 51,594 Ar a month [29]. Monthly revenue per household member was calculated by dividing the household’s monthly income by the household size. Quantitative data were analyzed using Microsoft Excel version 15.32 (Microsoft Corporation, USA).

### Qualitative Methodology

FGDs focused on assessing the motivation, expectations, and general attitude of a mobile phone–based savings and payment platform for skilled health care during pregnancy among clients.

#### Sampling

Criterion-based purposeful sampling was employed with the goal of obtaining information-rich data [30]. The criteria considered included women in reproductive age being pregnant at the time of sampling or having recently given birth, visiting a public health facility for ANC or postpartum care. As validity, meaningfulness and insights generated from qualitative inquiry have more to do with the information-richness of the cases selected and the observational capabilities of the researcher, sample size was determined on the basis of saturation of themes across FGDs [31]. We included 2 to 5 participants per group discussion. Women who took part in the questionnaire of the quantitative component of the study were excluded from participation in FGDs. If, upon consultation with local colleagues or upon piloting tools, we found that combining participants by a certain characteristic (economic status, age, etc) was impeding or undermining the quality of data collected, we altered the makeup of FGDs to be more sensitive to a particular dynamic.

#### Data Collection

Data collection occurred during the same time period as the quantitative survey (between November 29, 2017 and January 22, 2018). FDGs were led by a trained and experienced interviewer from the study team and were audio-recorded for analysis. FGDs were conducted in Malagasy, transcribed verbatim, and translated into English by 2 bilingual speakers. The qualitative instrument contained 15 open-ended questions relating to barriers to skilled care, attitudes, and habits toward health care savings and motivation, expectations, and general attitude toward the MHW for health care during pregnancy. All data collection activities took place at the health care facility in a separate room, ensuring confidentiality during the interview and discussions.

#### Data Analysis

Moreover, 2 of the authors conducted content analysis. A deductive approach was used to identify common themes, which were hand-coded and analyzed by the authors NM and ER. Each interview transcript was coded, and codes were grouped into categories on the basis of commonalities and patterns. Themes arising from the qualitative component were matched unto themes employed by the questionnaire survey such as finance-related issues during pregnancy, barriers toward saving, and potential benefits seen for the application of MHW [32].

#### Ethics Approval and Consent to Participate

Ethical clearance for the study was obtained from the Heidelberg University Hospital Ethics Committee (No. S-703/2017) and the study was approved by the Madagascan Ministry of Health. Written informed consent was obtained from all study participants before enrollment and participants were informed that participation would not affect their access to health care or the quality of care they receive. All participants were explicitly given the right to refuse participation.

## Results

### Quantitative Results

A total of 412 participants were included in the study. Table 1 shows the selected characteristics of study participants. The mean age of participants in the quantitative section was 25 years (range: 15-45 years). A total of 63.3% (261/412, 95% CI 58.6-68.0) of participants had at least attended the first year of secondary school and 94.1% (386/410, 95% CI 91.8-96.4) were literate. At the time of the survey, 77.9% (321/412, 95% CI 73.9-81.9) and 22.1% (91/412, 95% CI 18.1-26.1) of women attended ANC or PNC, respectively. Moreover, 70.9% (292/412) of participants answered questions regarding both average household income and household size, allowing their poverty status to be calculated with regard to the extreme poverty line of US $1.90 per person per day (2011 value, adjusted for purchasing power parity, 1719.8 Ar per day, 2016 value). Among those participants with data on poverty status, 56.0% (164/292, 95% CI 50.8-61.2) lived in extreme poverty. A total of 89.2% (260/292, 95% CI 86.0-92.4) of households had an income of less than 400,000 Ar per month (equivalent to US $126). Despite the overall low-income level, 58.7% (203/346, 95% CI 53.5-63.9) of participants owned a television (TV), whereas 76.1% (265/348, 95% CI 71.6-80.6) of the households did not own a vehicle (ie, bicycle, moped, car or any other wheel drive). The majority of the participants’ households (289/405, 71.4%, 95% CI 67.0-75.8) had access to a source of electricity, that is, municipal electricity supply, solar or generator.

**Table 1:** Selected characteristics of study participants in the quantitative component.

Sociodemographic characteristics			Statistics, n (%)
**Age group in years (N=411)**			
	15-18		61 (14.8)
	19-21		84 (20.4)
	22-25		108 (26.3)
	26-30		82 (20.0)
	31-35		39 (9.5)
	>35		37 (9.0)
**Marital status (N=412)**			
	Single		22 (5.3)
	Married		269 (65.3)
	Partnership		121 (29.4)
	Widowed		0 (0)
	Divorced		0 (0)
**Number of children (N=412)**			
	0		152 (36.9)
	1		130 (31.6)
	2		76 (18.4)
	3		30 (7.3)
	4		11 (2.7)
	>4		13 (3.2)
**Highest school attended (N=412)**			
	None		8 (1.9)
	Primary		143 (34.7)
	Secondary		166 (40.3)
	Bac^a^		53 (12.9)
	Higher		42 (10.2)
**Occupation (N=406)**			
	None		173 (42.6)
	Merchant		102 (25.1)
	Farmer		43 (10.6)
	Teacher		10 (2.5)
	Other		78 (19.2)
**Income and wealth assets**			
	**Monthly household revenue band (N=351)**		
		<50,000 Ar^b^	44 (12.5)
		50,000-100,000 Ar	53 (15.1)
		100,000-200,000 Ar	137 (39.0)
		200,000-400,000 Ar	79 (22.5)
		>400,000 Ar	38 (10.8)
	**Vehicle ownership (N=348)**		
		None	265 (76.1)
		Bicycle	33 (9.5)
		Moped	30 (8.6)
		Moped and bicycle	3 (0.9)
		Car	10 (2.9)
		Car and other vehicle	7 (2.0)
	**Television ownership (N=346)**		
		Yes	203 (58.7)
		No	143 (41.3)
	**Electricity at household (N=405)**		
		Yes	289 (71.4)
		No	116 (28.6)
**Mobile phone access and experience with Mobile Money**			
	**Mobile phone ownership (N=412)**		
		Personal phone	209 (50.7)
		Not personal, but family phone	122 (29.6)
		No personal or family phone	81 (19.7)
	**Knowledge about MM^c^ (N=412)**		
		Never heard about MM	39 (9.5)
		Knowing about MM without having used it	226 (54.9)
		Having ever used MM	147 (35.7)

^a^Bac: Baccalauréat (A-level, European Qualifications Framework Level 4).

^b^Ar: Ariary.

^c^MM: Mobile Money.

#### Use of Mobile Phones and Mobile Payment Services

A total of 80.3% (331/412, 95% CI 76.5-84.1) of participants made regular use of a mobile phone (50.7% and 29.6% owned or had access to a mobile phone within the immediate family, respectively). Out of 4 mobile phone providers active in Madagascar, 96.3% (319/331, 95% CI 94.3-98.3) of women with phone access were registered with either one or both of the 2 most popular operators and 18.7% (62/331, 95% CI 14.5-22.9) of these were registered with more than one provider at the time of the survey. Almost all respondents had heard about mobile payment services (90.5%, 373/412, 95% CI 87.7-93.3). However, only 35.7% (147/412, 95% CI 31.1-40.3) of these women had ever used it before. Of those, 67.4% (93/138, 95% CI 59.5-75.3) reported to use MM services solely for receiving funds from or sending funds to relatives and friends or for making cash withdrawals at registered agents. Surprisingly, 26.1% (36/138, 95% CI 18.7-33.4) used the technology additionally or solely for saving, whereas at the time of the survey, none of the phone operators offered a dedicated mobile savings account. Payment of bills and services using MM was uncommon (3.6%, 5/138, of MM users). One quarter of MM users reported ever having sent money to relatives for health care and only 1.4% (2/139) had ever used MM to pay for drugs or services at a health care facility. A total of 3 quarter of the users (75.0%, 99/132, 95% CI 67.6-82.4) reported to use MM technology between 1 and 4 times per month.

#### Savings Habits and Mechanisms

**Figure 1:**
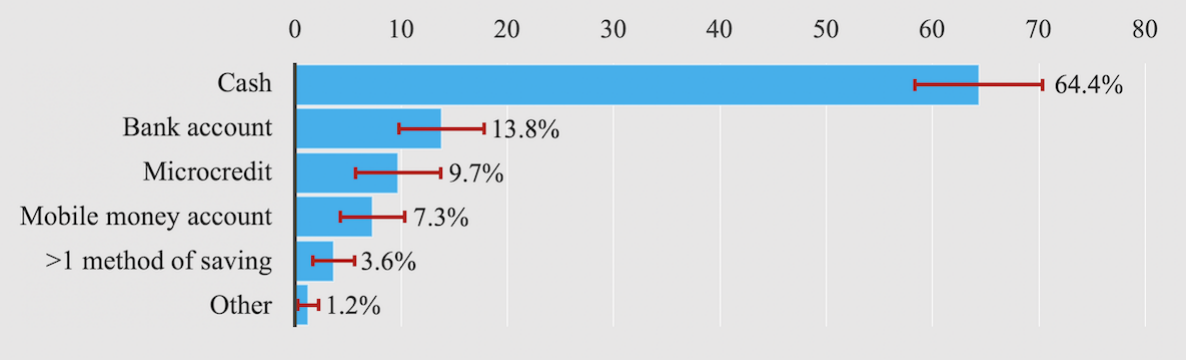
Saving methods among pregnant women and new mothers in Antananarivo (Atsimondrano and Renivohitra districts), Madagascar. N=247. Error bars represent 95% CIs.

Despite a low average household income, when asked if they put money aside in the form of savings, 59.3% (243/410, 95% CI 54.5-64.1) said yes, whereas 40.7% said they did not. The most prevalent method was household cash savings (64.4%, 159/247, 95% CI 58.6-70.2; Figure 1). Of those saving money, 55.4% did so on a regular basis, whereas 44.6% only saved on special occasions. 69.5% (171/249, 95% CI 63.7-75.3) of participants saved for delivery or newborn care. Only 34.4% (85/247, 95% CI 28.5-40.3) of participants who did save used formalized savings mechanisms such as a bank or MM account or used a microcredit scheme especially for saving.

#### Intended Place of Delivery

A total of 33.8% (132/391, 95% CI 29.1-38.5) of pregnant women stated the intention to deliver in a basic health care facility, whereas 52.2% (204/391, 95% CI 47.2-57.2) stated an intention to deliver in a community hospital. Only 5.1% (20/391, 95% CI 2.9-7.3) of women planned to deliver at home. Overall, 5.6% (22/391, 95% CI 3.3-7.9) of respondents mentioned the intention to deliver in a private hospital.

#### Media Access and Consumption Habits

**Figure 2:**
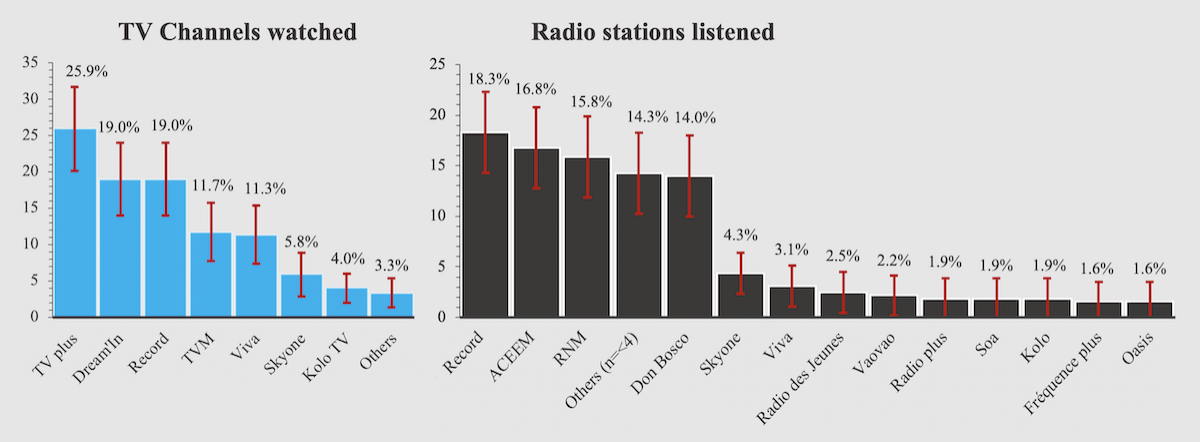
Media consumption among pregnant women and new mothers in Antananarivo (Atsimondrano and Renivohitra districts), Madagascar. TV: N=228. Radio: N=277. Error bars represent 95% CIs.

**Figure 3:**
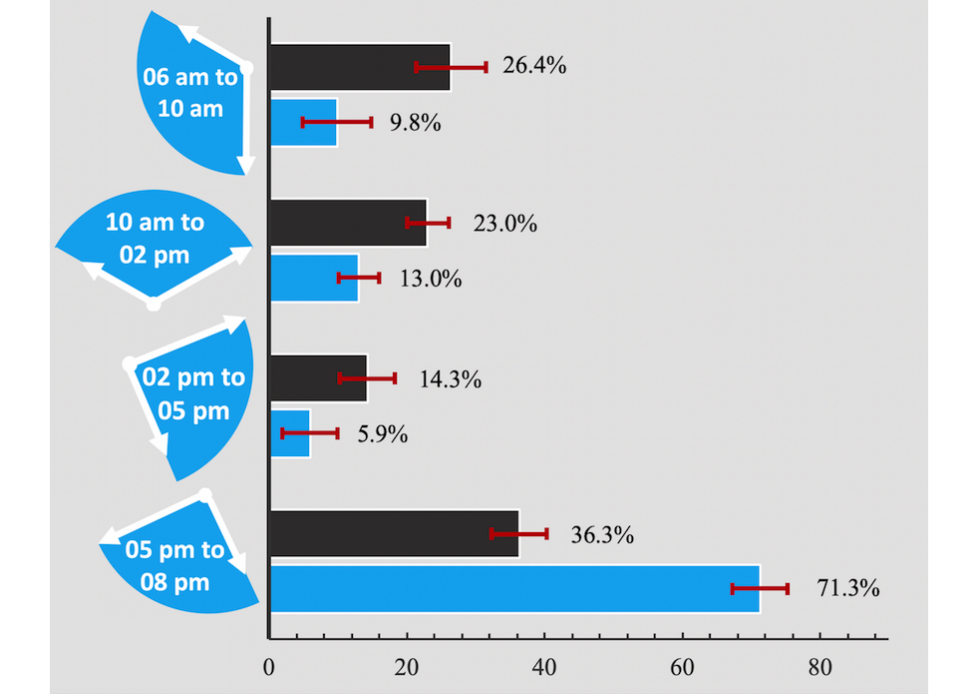
Day time of media consumption among pregnant women and new mothers in Antananarivo (Atsimondrano and Renivohitra districts), Madagascar. TV: N=228 (blue). Radio: N=277 (black). Error bars represent 95% CIs.

A total of 67.4% (277/411, 95% CI 62.9-71.9) and 55.5% (228/411, 95% CI 50.7-60.3) of the participants reported having regular (at least once a week) access to radio and TV, respectively (Figure 2). They cited 34 radio stations and 12 TV channels that they listened to or watched, with the most popular radio station (Radio Record) and TV channel (TV plus) being cited by 18.3% (50/274, 95% CI 14.1-22.5) and 25.9% (59/228, 95% CI 20.7-31.1) of the women, respectively. Reported times of radio listening were spread across the day, whereas TV watching mainly took place during the evening hours from 5 pm to 8 pm (Figure 3). Only 20.6% (84/408, 95% CI 16.7-24.5) of the respondents were connected to the social media platform Facebook and most of the users (82.1%, 69/84, 95% CI 73.9-90.3) consulted the platform on a mobile device.

### Qualitative Results

#### Barriers to Utilizing Skilled Care

A total of 24 participants attended 6 sessions of FGDs. The average age of the participating women was 23.3 years (range: 14-39). FGDs revealed that major barriers to skilled care included inadequate information (particularly on the medical necessity, health benefits, availability, and costs of maternal health care services), financial obstacles, and cultural aspects. Some respondents explained that even minor expenses (ie, for iron and folic acid supplements) prohibited pregnant women from seeking institutional care. Several of the respondents stated that fear of unexpected and expensive hospitalization in case of complications detected during a routine ANC visit made them hesitate to seek skilled care. One pregnant woman stated her thoughts:

I am wondering if It will be a normal birth or if it will require surgery. I see the other [women] and I become anxious: When the delivery is normal, we don’t have any problem and can pay for it. But if it happens to be a complicated delivery, we need an operation and spend a lot of money.

Pregnant woman

Another aspect contributing to financial obstacles for seeking skilled care was nonconsistent prices at the provider level. Participants stated that prices for drugs and services vary over time and among individual patients (including among patients treated at the same public-sector health facility), thereby adding to the unpredictability of costs. Time constraints caused by the necessity to generate income to cover daily costs of living were cited as an obstacle for women who did not have a regular job, as explained by the following respondent:

They [unemployed women] are busy searching for income. Instead of coming for an antenatal care visit they have to work. They don’t have time to spend here [at the health center].

Young mother

Overall, it was agreed that costs for home deliveries without qualified birth attendance or traditional birth attendance were more predictable and generally cheaper than facility-based births and were the preferred alternative for delivery if savings appeared to be insufficient. Cultural aspects including low prioritization of maternal health care and general skepticism toward skilled care were further commonly cited obstacles:

I am afraid of going to hospital because you have mainly students there and you are used for their op [surgical] training, so people are really afraid and don’t go there anymore. They are all trainees and that is scary. And even for small problems – for instance you [the birth process] take longer, they are not patient to wait and send you directly to the operating room.

Pregnant woman

#### Savings Habits and Mechanisms

Respondents mostly saved on a weekly or monthly basis by putting aside a part of their cash earnings in a savings box at home. However, participants acknowledged that expenses such as school fees, funeral costs, or other substantial unforeseen financial crises within the family frequently endangered their health care savings:

When I will be in the 8th month of my pregnancy, I will begin to buy baby clothing. I don’t save but hope - I really pray for it - that the baby will come at the time my husband will receive his salary. We do not have the possibility to save. But when I save and there is nothing else left, I break the savings box...I really pray that I will deliver the baby around pay day.

Pregnant woman

A major motivation to use formalized savings mechanisms was to preclude savings from being used other than for the intended savings goal. However, the majority of FGD participants expressed only low levels of frustration when savings goals were not met. Some respondents stated that borrowing money for health care from a relative or friend was culturally accepted and common under such circumstances:

I bring my savings to the post office. Because if I keep it home, I will use it. When I don’t have [money] I have no choice but using it. Now when the delivery date is approaching, I will take it in person because they [the financial institution] don’t give it to someone else. Only to the owner.

Pregnant woman

#### Attitude and Expectations Toward a Mobile Phone–Based Health Care Savings Platform

FGD participants perceived the concept of MHW for maternal health care as generally beneficial. A particular value was seen in conditional funds that can only be spent on maternal health care. However, respondents emphasized that inaccurate or incomplete information about benefits or restrictions of the service could inhibit its acceptance and use by the target population. A comprehensive sensitization campaign was deemed crucial by respondents for a successful implementation of the service and should certainly include personal contact between program agents and users. Ease of use of MHW and personal customer support by a health worker or dedicated agent were mentioned by some respondents to be essential for acceptance by the population:

Once the money is in it [the platform] you are not allowed to take it. This point is what attracts me because the saving remains untouched, not like with savings box. That is the money dedicated to face delivery. Or to buy drugs.

Pregnant woman

## Discussion

### Principal Findings

This study revealed 3 salient findings. First, the target population in the peri-urban areas of Antananarivo is relatively privileged with regard to the Madagascan average, with relatively high rates of secondary school attendance and media access and almost universal literacy. Although more than half of the study group falls below the World Bank absolute poverty line of US $1.90 per day, this proportion is still well below the average for the entire country. Second, although the concept of MM was familiar to almost all respondents, and mobile phone access was widespread, only a minority were currently using the technology, predominantly for sending and receiving money between friends and relatives but less commonly as a savings tool. The use of MM to pay for health care services was infrequent. Third, in this study’s FGDs, many participants raised the issue of financial limitations to accessing ANC and PNC resulting from OPP and opportunity costs. Participants reported difficulties in saving for health care and reported a variety of informal tools to help them save. Interest in a mobile phone–based savings and payment platform such as the MHW was raised by nearly all participants. To our knowledge, this study includes the first qualitative research analyzing general and financial barriers of access to skilled health care for pregnant women in the capital of Madagascar.

Our findings are generally consistent with data of the *Enquête Nationale sur le Suivi des indicateurs des Objectifs du Millénaire pour le Développement* (ENSOMD—national survey on the progress toward the Millennium Development Goals), conducted by the Madagascar National Institute of Statistics in 2012 to 2013. This report listed financial restrictions as a major obstacle toward health care for pregnant women in Antananarivo [20]. However, conversely, issues about fear of lack of drugs and absence of health care staff were not raised by the participants of our FGDs. This might reflect the urban and peri-urban setting of this study resulting in lower staff absenteeism (because of high visibility of the health centers) and better supply routes for drugs and materials. Another potential explanation could be the difference in location of interviews, whereas this study was conducted in public-sector health facilities, ENSOMD interviews were held in pregnant women’s homes, potentially reducing barriers to reporting system deficiencies.

In addition to the ENSOMD survey, only 2 other studies have analyzed obstacles to health care access for pregnant women in Madagascar, though neither focused on financial birth preparedness. One study investigated the available sources of funds utilized to pay for obstetric emergency care in a hospital in Mahajanga, a town in northwestern Madagascar [5]. One in 6 households included in the study was able to pay the costs incurred during hospital admission from routine income or savings. Borrowing money from family and friends was needed in most cases to complete payments for maternal health care services. These findings are in line with our results, albeit for a different urban area of Madagascar. However, the study was limited in scope (including 103 mothers in a single hospital) and only focused on emergency obstetric and neonatal care in a referral hospital rather than focusing on financial preparation. The second study, conducted in Fort Dauphin, an urban center in the southeast part of the island [33], identified lack of knowledge (as observed in this study), risky practices, delay in seeking medical care, and family and community expectations as major obstacles. We suspect that these factors are rather specific to the region under study, which is known to be one of the poorest areas of the country and with high levels of influence exerted by traditional social norms [33,34]. A further obstacle—the distance of travelling to the next health care facility—was not an important barrier among the population in this study, likely because Antananarivo is densely populated and the average distance to the nearest health care facility is relatively short [35].

The results show that despite national policies promising free ANC and delivery in Madagascar, in practice, the majority of obstacles to ante, perinatal, and postpartum health care access cited by the participants were of a financial nature [21]. High and highly variable financial contributions, that is, for complementary clinical diagnoses or the treatment of complications, represent a tremendous burden for poorer women and can create fear of engaging with formal health care. Not a single facility observed in this study had price information visibly displayed within the center. Indeed, among all our respondents from FGDs, a considerable lack of knowledge about prices for health care services was identified. Indeed, services that were free of charge were unknown, and prices for specific services were unclear. The women in this study feared pregnancy-related complications and the need for unexpected additional care because of the threat of impoverishment because of high health care expenditures.

Unpredictable pricing of health care services can have a number of different origins, including the following: discontinuous availability of drugs and consumables that are donated and thus free of charge at point of care, prices set by individual health care centers and outside of official regulations, as well as costs differing by the individual health worker providing care. In all these cases, the lack of transparency and absence of predictable and clearly communicated costs of pregnancy-related care are likely to discourage pregnant women from taking advantage of health care services and motivate them to search for alternative care outside of the formal sector. Transparency of prices was mentioned as a critical issue by respondents to plan for delivery, and it needs to be taken into consideration by policy makers or program managers considering the implementation of an MHW in Madagascar.

Rotating Savings and Credit Associations or community savings groups, known in Madagascar as “tontines” [36,37], were not cited by respondents, and they do not seem to play a major role for health care financing in the study setting. Similarly, the role of the Madagascan *Fonds d’Équité* (an “equity fund” established by the Ministry of Health to finance health expenditures for the poorest section of the population) was suggested to be modest. One reason may be reluctance to be stigmatized as in need of financial help, as to benefit from these funds, patients need to prove their neediness and register at the community level (Malagasy: Fokontany) [38]. Taken together, our findings underline the importance of identifying and implementing novel means of health care financing to eliminate or mitigate OPPs, facilitate financial birth-preparedness, and reduce the risk of health shocks. MHW may play an important role for inclusion in health care in LMICs [39].

### Limitations

This study has several limitations. First, we recruited study participants in randomly selected public-sector health facilities, and the questionnaire and FGDs occurred in this setting. This means that all the participants were privileged by already having access to public health care. We decided for this method of sampling as it gave us the opportunity to include a representative number of pregnant women and young mothers and as this group of women will be included during a planned intervention study to examine the impact of an MHW on skilled birth attendance in Madagascar. Future research should include women without preexisting access and women who had decided not to engage with the public-sector health facilities, who are likely a poorer subpopulation. Second, this study focused on the urban and peri-urban areas of Antananarivo. This setting was chosen on the basis of key informant advice regarding better mobile phone network coverage and high levels of mobile phone usage in the area. Rapidly increasing mobile phone usage in other, including rural, areas of Madagascar may make the country-wide application of MM-based savings systems feasible in the near future. However, the chosen setting may have led to sampling bias. Inhabitants of the study area, for instance, were on average of higher socioeconomic status relative to the country average. Further research is needed to determine the acceptability of an MHW in rural and noncapital urban areas. Third, this study only evaluates perception of usefulness and practicability of the MHW by pregnant women. However, as widely described for several resource-low settings, successful implementation and sustainability is not only solely dependent on the acceptance by the target population but also by other stakeholders such as community heads, health care professionals, and health officials. Additional research on the acceptance of the tool by other health care stakeholders is therefore needed. Fourth, solutions for demand-side affordability alone will not be sufficient to deliver adequate antenatal and obstetric care. Concurrent improvements in health care infrastructure and staff skills will also be necessary. Finally, our results may not apply to other countries. Nevertheless, given the current gap in the literature, our results may be informative to researchers and policy makers in similar settings in SSA.

### Conclusions

Financial obstacles are a major cause of limited access to skilled maternal health care in low-resource settings and especially in urban Madagascar. A mobile phone–based savings and payment platform for skilled health care during pregnancy was perceived as a useful tool to reduce financial barriers among women in the capital of Madagascar. Key factors that may contribute toward a successful implementation of MHW among this population include the following: (1) a high willingness to save, (2) broad mobile phone usage, (3) cultural acceptance of a mobile payment and savings tool, and (4) the perceived usefulness of the system by pregnant women. However, to enhance the access toward maternal health care by the tool, a number of financial obstacles need to be tackled. Out-of-pocket costs of basic treatment were high, and transparency about free services and prices was inadequate. A culturally sensitive communication and sensitization strategy and comprehensive technical support will be essential to fill the existing gap of knowledge and overcome cultural restrictions. Future research must determine whether and how a mobile phone–based payment platform can enhance access to improve maternal health care delivery and ultimately maternal health outcomes. 

## Abbreviations

ANC: antenatal care
ENSOMD: Enquête Nationale sur le Suivi des indicateurs des Objectifs du Millénaire pour le Développement (national survey on monitoring the indicators of the Millennium Development Goals)
FGDs: focus group discussions
LMICs: low- and middle-income countries
MHW: Mobile Health Wallet
MM: Mobile Money
OPP: out-of-pocket payment
PNC: postnatal care
SSA: sub-Saharan Africa
TV: television
