# Metadata Correction: A Mobile Health Wallet for Pregnancy-Related Health Care in Madagascar: Mixed-Methods Study on Opportunities and Challenges

**DOI:** 10.2196/16190

**Published:** 2019-10-14

**Authors:** Nadine Muller, Peter Martin Ferdinand Emmrich, Elsa Niritiana Rajemison, Jan-Walter De Neve, Till Bärnighausen, Samuel Knauss, Julius Valentin Emmrich

**Affiliations:** 1 Heidelberg Institute of Global Health Medical Faculty and University Hospital University of Heidelberg Heidelberg Germany; 2 Department of Infectious Diseases and Pulmonary Medicine Charité-Universitätsmedizin Berlin, corporate member of Freie Universität Berlin, Humboldt-Universität zu Berlin, and Berlin Institute of Health Berlin Germany; 3 Biosciences Eastern and Central Africa Hub International Livestock Research Institute Nairobi Kenya; 4 Department of Global Health and Population Harvard T.H. Chan School of Public Health Boston, MA United States; 5 Africa Health Research Institute Mtubatuba, KwaZulu-Natal South Africa; 6 Department of Experimental Neurology and Center for Stroke Research Charité-Universitätsmedizin Berlin, corporate member of Freie Universität Berlin, Humboldt-Universität zu Berlin, and Berlin Institute of Health Berlin Germany

In “A Mobile Health Wallet for Pregnancy-Related Health Care in Madagascar: Mixed-Methods Study on Opportunities and Challenges” (JMIR Mhealth Uhealth 2019;7(3):e11420), authors Knauss and Emmrich should have been denoted as having contributed equally to the paper but were not. A footnote clarifying equal contribution has now been added to each of the aforementioned authors.

The correction will appear in the online version of the paper on the JMIR website on October 14, 2019, together with the publication of this correction notice. Because this was made after submission to PubMed, PubMed Central, and other full-text repositories, the corrected article has also been resubmitted to those repositories.

